# Multicancer early detection in a cohort of patients with confirmed and suspected cancer by measuring plasma amino acid cross sections with the Enlighten test: MODERNISED protocol

**DOI:** 10.1136/bmjopen-2025-108220

**Published:** 2025-11-04

**Authors:** Sam Wilding, Victoria Goss, Wesley Sukdao, Zaed Hamady, Joanne Lord, Adam Coleman, Catherine Pointer, Jocelyn Walters, Will Herbert, Katy Mclaughlin, Robert Waugh, Nicola Irvine, Tom Oliver, Irene Soulsby, Julie Hooper, Simon J Crabb, Gareth Griffiths, Emma Yates, Andrew Davies

**Affiliations:** 1 Cancer Research UK Southampton Clinical Trials Unit, University of Southampton and University Hospital Southampton NHS Foundation Trust, Southampton, Hampshire, UK; 2 Proteotype Diagnostics, Cambridge, UK; 3 University Hospital Southampton NHS Foundation Trust, Southampton, UK; 4 Southampton Health Technology Assessments Centre, University of Southampton, Southampton, Hampshire, UK; 5 Cancer Research UK/National Institute for Health and Care Research, Southampton Experimental Cancer Medicine Centre, University of Southampton, Southampton, UK; 6 Patient and Public Representative, Southampton, UK

**Keywords:** Early Detection of Cancer, Cancer, HISTOPATHOLOGY

## Abstract

**Introduction:**

Detecting cancer earlier improves treatment options and long-term survival. A multicancer early detection test that reliably picks up early-stage cancer would potentially save lives and reduce the cost of treating cancer. One promising candidate is the *Enlighten* test, which applies machine learning to plasma amino acid concentrations to detect cancer. In a cohort of 77 patients recently diagnosed with breast, colorectal, pancreatic or prostate cancer, 60 (78%) were detected by the test (sensitivity), with no false positives in 20 healthy controls. The MODERNISED study will further develop the Enlighten test to detect 10 different cancers by adding bladder, lung, melanoma, oesophageal, ovarian and renal cancer to the test.

**Methods and analysis:**

MODERNISED (ISRCTN17299125) is a multicentre prospective, non-interventional, case–control study. We aim to recruit 1000 adult participants with a recent cancer diagnosis, 250 adult participants with symptoms of cancer where a cancer diagnosis was ruled out by the National Health Service (NHS) standard of care and 100 healthy adult volunteers. Cancer tissue of origin (ToO) will include bladder, breast, colorectal, lung, melanoma, oesophageal, ovarian, pancreatic, prostate and renal. Participants in the two non-cancer cohorts who are later diagnosed with cancer will be moved to the cancer cases cohort. The primary aim is to train and validate a machine learning algorithm to detect cancer, which will be evaluated by AUROC. Secondary aims include training and validating an algorithm to predict ToO and stage of cancer, exploring differences in performance by demographics and estimating how sensitivity varies across specificity cut-offs of 95%, 99% and 99.9%. These results will provide a statistically powered estimate of how well the Enlighten test can discriminate between individuals with and without cancer, which can then be validated for clinical use in further research.

**Ethics and dissemination:**

This study is sponsored by University Hospital Southampton NHS Foundation Trust and has been approved by the Health Research Authority and Health and Care Research West Midlands (24/WM/0234). Results will be presented at scientific meetings and published in international peer-reviewed journals. Lay summaries of study progress and findings will be published on the Southampton Clinical Trial Unit’s website.

**Trial registration number:**

ISRCTN17299125.

**STRENGTHS AND LIMITATIONS OF THIS STUDY**

- Multicancer early detection (MCED) tests are proposed as tools for improving the detection of cancer in the early stages, but most use cell-free DNA technology, which is expensive and has demonstrated poor sensitivity for stage I and stage II cancers.
- Amino acids may be more successful in detecting early-stage cancer because immune responses are stronger during the early stages of cancer.
- This study will use the Enlighten test, a new candidate MCED that applies machine learning to amino acid level patterns and has a sensitivity of 76% and specificity of 100% in a cohort of 72 cancer patients and 20 healthy controls.
- This study will retrain and evaluate the Enlighten test in newly diagnosed cancer patients, symptomatic patients who had cancer ruled out and healthy volunteers across southern England.
- Reliable and cheap MCED tests would potentially save lives and reduce the cost of treating cancer, but further validation in future research will be required before clinical implementation.

## Introduction

 Cancer is one of the leading causes of death worldwide, with 149 022 deaths attributed to cancer in England and Wales across 2023.^1^ Detecting cancer earlier improves the number of treatment options available and long-term survival.^2^ In 2019, the National Health Service (NHS) in the UK committed to increasing the proportion of cancers that are diagnosed at stage I or II.^3^ There has been minimal change in the proportion between 2019 (53.4%) and 2022 (54.8%), despite an increasing number of people referred for cancer diagnostics.^4 5^ Additional tools are required to detect cancer at an early stage, which would improve the proportion of cancers diagnosed at an earlier stage, improve patient outcomes and reduce the cost burden on the system.

### Multicancer early detection (MCED) tests

MCEDs, tests that analyse a ‘liquid-biopsy’ such as blood, urine, saliva or stool and test for the presence of multiple cancers in a singular sample, are being proposed as a complement to traditional cancer diagnosis pathways to improve early detection.^6^ A 2024 systematic review found 36 studies evaluating six different MCEDs, with most having a high risk of bias.^7^ There are no randomised studies demonstrating that using MCEDs leads to an increase in the proportion of cancers diagnosed at stage I or II. The majority of MCEDs that have published data available are using cell-free DNA (cfDNA) or circulating-tumour DNA (ctDNA), which are fragments of DNA released into the bloodstream that may possess alterations indicative of cancer.^8^

The degree of mutations present within cfDNA and ctDNA increases as cancer progresses, so this approach may not be successful if the aim is to detect cancers at stage I or II, where the amount of material shed into the bloodstream is below the limit of detection.^9^ The largest evaluation of an MCED in the UK, the SYMPLIFY study, used the GRAIL test on 5461 symptomatic patients referred for urgent investigation for a possible gynaecological, lung or gastrointestinal cancer. The sensitivity for cancer detection was much lower at stage I (24.2%) than at stage IV (95.3%).^10^ This is a common trend among cfDNA MCEDs. One meta-analysis found a pooled sensitivity of 50.3% for stage I or II versus 77.4% for stage III or IV.^11^ To increase early cancer detection, alternative techniques are required.

### The Proteotype Diagnostics Enlighten test

Proteotype Diagnostics (PD) have developed an alternative approach to MCED testing. Instead of measuring material released from tumours (cfDNA or ctDNA), amino acid concentrations are measured in plasma in the Enlighten test, which are affected by the immune response. Immune responses are stronger during the early stages of cancer^12^ and therefore may be used to improve the detection of stage I and II cancers. Machine learning (an ensemble subspace discriminant classifier) was applied to classify tested samples to cancer or non-cancer in a retrospective observational study of 77 cancer patients (breast, colorectal, pancreatic and prostate) and 20 healthy volunteers. A sensitivity of 78% at 100% specificity was achieved using Enlighten, and concentrations were further differentiated in early-stage cases.^13^

Southampton Experimental Cancer Medicine Centre (ECMC) responded to an open call for partnerships with PD to develop a study further evaluating the Enlighten test. Southampton Clinical Trials Unit (SCTU) then developed a research proposal with PD and ECMC as partners, which is academically sponsored by University Hospital Southampton Trust and jointly funded by the National Institute for Health and Care Research and the Office for Life Sciences (ref NIHR207538).

The primary aim of this study is to train a machine learning classifier to use amino acid concentrations from the Enlighten test to detect cancer (bladder, breast, colorectal, lung, melanoma, oesophageal, ovarian, pancreatic, prostate and renal) in comparison to adult participants with symptoms of cancer where a cancer diagnosis was ruled out and healthy volunteers. Participants will be randomised, with 75% used to train the classifier and 25% used to validate the classifier. Secondary aims include:

1. Training and validating a classifier to distinguish cancer patients from adult participants with symptoms of cancer, where a cancer diagnosis was ruled out.
2. Training and validating a classifier to distinguish cancer patients from healthy controls.
3. Evaluating discrimination and sensitivity by tissue of origin (ToO), stage, age, sex at birth and availability of a UK cancer screening programme.
4. Training and validating a classifier to predict ToO.
5. Training and validating a classifier to predict cancer stage.

## Methods and analysis

### Study design

MODERNISED (ISRCTN17299125) is a multicentre prospective, non-interventional, case–control study recruiting 1000 participants with known cancer, 250 under investigation for cancer where cancer was ruled out, and 100 healthy volunteers. Participants will be recruited from five secondary care sites and healthy volunteer register banks, or hospital or university staff in England. Recruitment began in January 2025. Recruitment is underway, and version 2 (01 April 2025) is the latest version of the study protocol at the time of submitting this manuscript. Important protocol modifications are reported to relevant parties as part of the SCTU’s standard operating procedures.

### Study participants

**Figure 1:**
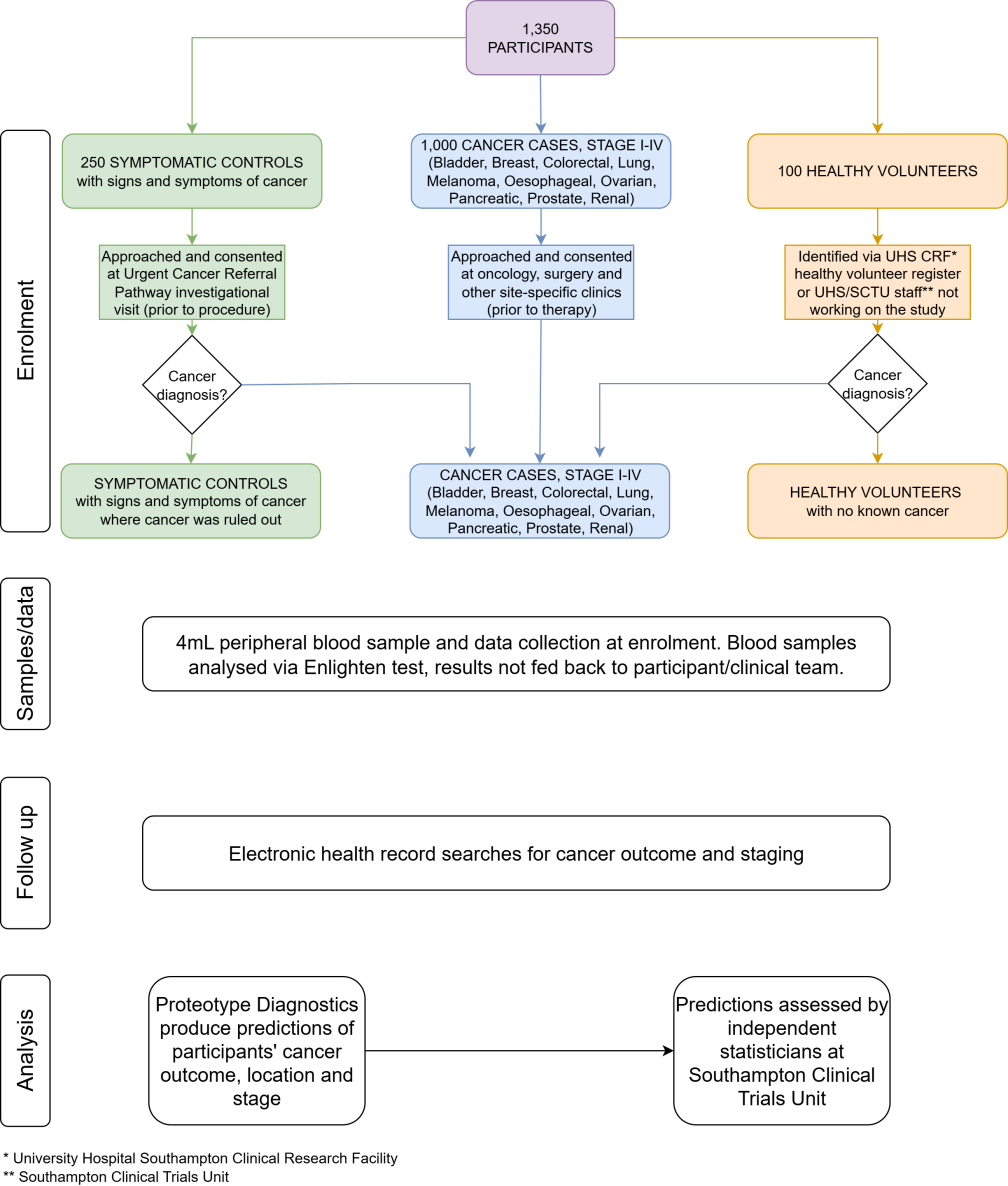
MODERNISED study workflow. CRF, clinical research facility; SCTU, Southampton Clinical Trials Unit; UHS, University Hospital Southampton.

The first cohort, ‘cancer cases’, will consist of 1000 adults recently diagnosed with one of the 10 cancers, recruited from secondary care sites prior to treatment. The second cohort, ‘symptomatic controls’, will consist of 250 adults either currently or previously under investigation for one of the 10 cancers, recruited from secondary care sites, who are found to be cancer-free following normal diagnostic pathways. Participants in this cohort who are found to have cancer during follow-up will be moved to the cancer cases cohort. These participants will be reached via screening of clinic lists and approached during routine hospital visits for symptom investigation, or by phone call to invite them to attend an enrolment visit. The third cohort, ‘healthy volunteers’, will consist of 100 adults with no known cancer and no active symptoms indicative of cancer, recruited from volunteer groups at secondary care sites, or University and hospital staff if recruitment is slower than anticipated. These participants will be reached by advertisement of the study in email newsletters and invited to attend recruitment clinics. Participants in this cohort who are found to have cancer during follow-up will be moved to the cancer cases cohort. Full inclusion and exclusion criteria can be found in table 1. The study workflow is outlined in figure 1.

**Table 1:** Inclusion and exclusion criteria

Entry arm	Inclusion criteria	Exclusion criteria
All	1. Age 18 years or older. 2. Able to provide a written informed consent. 3. Willing to provide a 4 mL blood sample at the time of enrolment.	6. Pregnancy (by self-report of pregnancy status), or pregnancy within the last 12 months. 7. Current febrile illness. 8. Infection requiring hospitalisation within 30 days prior to blood draw. 9. Acute exacerbation or flare of an inflammatory condition requiring escalation in medical therapy within 14 days prior to blood draw. 10. Undergone a surgical procedure (including diagnostic biopsy) within 14 days prior to blood draw. 11. Undergone a blood transfusion within 30 days prior to blood draw. 12. Recipient of organ transplant or prior allogeneic stem cell transplant. 13. Poor health status or unfit to tolerate a blood draw. 14. Oral or intravenous corticosteroid use in the past 14 days prior to blood draw.
Cancer cases	4. Have either of the following: **Confirmed cancer diagnosis of** breast, melanoma, colorectal, lung, prostate, pancreatic, ovarian, oesophageal, renal or bladder cancer (any stage I–IV) **within 90 days prior to or up to 90 days after study blood draw**, based on assessment of a pathological specimen (including, but not limited to, biopsy from primary tumour site, lymph node or metastatic lesion, or cytology specimen). Or **a high suspicion for a cancer diagnosis of** breast, melanoma, colorectal, lung, prostate, pancreatic, ovarian, oesophageal, renal or bladder cancer by clinical and/or radiological assessment, with *planned* biopsy or surgical resection to establish a definitive diagnosis within 3 months (90 days) *after* study blood draw.	15. Currently receiving, or ever received, any of the following therapies to treat their current cancer: surgical management of the cancer beyond that required to establish the cancer diagnosis; local, regional or systemic chemotherapy, including chemoembolisation; targeted therapy, immunotherapy, including cancer vaccines; hormone therapy or radiation therapy. 16. Known prior diagnosis of cancer, per guidance in protocol, separate from the confirmed or suspected cancer diagnosis associated with study enrolment. **Exception**: subjects with a history of non-melanoma skin cancer that has been effectively and exclusively managed by local or focal therapies, such as surgical resection, radiation therapy, cryotherapy or topical therapy, are eligible to enrol. 17. Cancer recurrence. 18. Synchronous malignancies.
Symptomatic control	**5**. Have either of the following: **Symptomatic of** breast, melanoma, colorectal, lung, prostate, pancreatic, ovarian, oesophageal, renal or bladder cancer, **but determined to be cancer-free** by clinical and/or radiological assessment and/or assessment of a pathological specimen (including, but not limited to, biopsy from suspected primary tumour site, lymph node or suspected metastatic lesion, or cytology specimen)—for a list of symptoms please refer to online supplemental file 1. Or **an initial high suspicion for a cancer diagnosis of** breast, melanoma, colorectal, lung, prostate, pancreatic, ovarian, oesophageal, renal or bladder that was **ruled out** 90 days after study blood draw.	19. Known current or prior diagnosis of cancer, per guidance in protocol. **Exception**: subjects with a history of non-melanoma skin cancer (eg, Basal Cell Carcinoma or Squamous Cell Carcinoma) that has been effectively and exclusively managed by local or focal therapies, such as surgical resection, radiation therapy, cryotherapy or topical therapy, are eligible to enrol.
Asymptomatic control		20. Known current or prior diagnosis of cancer, per guidance in protocol. **Exception**: subjects with a history of non-melanoma skin cancer that has been effectively and exclusively managed by local or focal therapies, such as surgical resection, radiation therapy, cryotherapy or topical therapy, are eligible to enrol.

### Baseline data collection

Clinic and MDT lists will be screened regularly (minimum two times per day) to identify cancer cases referred to relevant clinics. Clinic and 28-day faster diagnosis pathway lists will also be used to identify individuals referred to secondary care by their general practitioner for symptoms, which indicate that they may have one of the 10 cancers being investigated. Healthy volunteers are recruited from registers where they have consented to be approached for involvement in research and will be contacted by the research team. If recruitment of healthy volunteers is challenging, the protocol allows for members of staff at the University Hospital Southampton and the University of Southampton to be approached. Those providing informed consent will be invited to a baseline visit (T_0_).

At the baseline visit, eligibility will be confirmed with a trained and delegated healthcare professional, who will also collect medical history, concomitant medications, demographics and the Index of Multiple Deprivation. Pseudonymised data will then be entered into Medidata’s Rave Electronic Data Capture, which can then only be accessed by allocated staff at SCTU. Participants will then have 4 mL of blood drawn into an EDTA tube. Any adverse events related to blood collection will be reported to the SCTU and summarised in the final study publication. All blood samples will be posted overnight Monday to Thursday at ambient temperature using Royal Mail Safeboxes (or equivalent) to the University Hospital Southampton NHS Foundation Trust, Wessex Investigational Sciences Hub (WISH). The Enlighten assay will be performed by Southampton ECMC-trained laboratory personnel housed in the WISH laboratory, and pseudonymised assay results (participant identifier and amino acid readings) will be transferred securely to PD to be converted to amino acid concentrations.

### Outcome measurement

Cancer outcome and staging will be retrieved from the Electronic Health Record (EHR) via local hospital research staff. This will be triggered for each participant once 3 months have passed since informed consent (T_1_), but data may be entered earlier where appropriate. Cancer diagnosis will be coded using ICD-10 to capture ToO, morphology and staging according to TNM (tumour, node, metastases) and cancer-specific staging system, as appropriate. Consent will be gained to collect further cancer diagnoses up to 2 years from participants’ enrolment.

### Model training and validation

Participants will be randomised in a 3:1 ratio with varying permuted blocks, balanced by cancer ToO and stage, into a model training (75% of participants) or model validation (25% of participants) set. The randomisation list will be pregenerated by a statistician at SCTU. Those in the model training set will have their cohort (cancer case, symptomatic control and asymptomatic control), age and sex shared with PD to train machine learning algorithms in combination with amino acid concentrations. PD will be blinded to the diagnostic status of participants in the model validation set.

Symptomatic controls and healthy volunteers may later be diagnosed with cancer (and therefore move into the cancer cases cohort). Randomisation will therefore only occur on three occasions during the study to reduce the impact of imbalance between building and validation sets. All participants and care providers will remain blinded. Participants will not be randomised more than once. The occasions are as follows: (1) when the amino acid concentrations have been measured for the first batch of 20 participants’ samples with complete data (to stress test processes). (2) When the T_1_ visit has occurred for 50% of the target participants in the breast, colorectal, prostate and lung cancer cohorts. (3) When the T_1_ visit has occurred for all participants, and all data have been returned.

### Machine learning training and validation

Machine learning techniques considered will include: random forests, support vector machines, ensemble subspace discriminant classifiers, XGBoost, neural networks and deep learning algorithms. The final modelling strategy will be informed by comparative predictive performance and computational cost. Triplicate values of the five amino acids (Cysteine, Reduced Cysteine, Lysine, Tryptophan and Tyrosine) will be used as input parameters (or combined as average readings), with potential additions of participant age and sex. Feature importance will be measured by Shapley values. Stratified cross-validation will be used to reduce overfitting.

One algorithm will predict diagnostic status (‘cancer’ or ‘no cancer’), a second will predict cancer ToO (bladder, breast, colorectal, lung, melanoma, oesophageal, ovarian, pancreatic, prostate and renal) and a third will predict cancer stage (I, II, III, IV). The trained algorithms will use amino acid concentrations (and potentially age and sex) to predict diagnostic status, ToO and cancer stage in the development and validation cohorts. These predictions will be provided to statisticians at SCTU to independently validate predictions against true status.

### Sample size calculation

A prediction model for a binary outcome (cancer yes/no status, prevalence 74%), 15 prediction parameters (five amino acids Cysteine, Reduced Cysteine, Lysine, Tryptophan and Tyrosine, potentially age and sex, and allowing for additional interactions in models), Nagelkerke’s r-squared of 20% and target shrinkage of 10% requires a minimum sample size of 916 (678 events) to reduce overfitting.^14^ Rounded up to 750 events and allowing a random 3:1 split between data available for model building and validation, the total required sample size is 1000 events, with 250 symptomatic controls, and 100 healthy volunteers, a total of 1350.

Of the 1350, the total sample size for validation would be 337, with 250 cancer cases, 62 symptomatic controls and 25 healthy volunteers. Because the distribution of the linear predictor derived from the prediction model is not known in advance, the anticipated Area Under the Receiver Operator Curve (AUROC) can be used to determine the required sample size for validation.^15^ Using this simulation approach with a target CI width of 0.1, the prediction model’s AUROC must be at least 0.84 to validate the model with sufficient accuracy with 337 individuals. As the pilot data demonstrated an AUROC of 0.98, it seems reasonable to assume an AUROC of 0.84 or higher can be achieved within this study.

### Analysis plan

Baseline medical history, concomitant medications, demographics and Index of Multiple Deprivation will be summarised using descriptive statistics by cohort. The number of symptomatic controls and healthy volunteers who are later diagnosed with cancer and moved into the cancer cases cohort will be presented.

PD will train machine learning algorithms for diagnostic status, ToO and cancer stage. The output for diagnostic status will be a continuous risk score. The output for ToO will be one of the 10 cancers involved in the study, and may include the percentage chance for each ToO. The output for cancer stage will be either stage I, stage II, stage III or stage IV, and may include the percentage chance for each stage. Statisticians at SCTU will produce an AUROC plot in the model training and validation groups for diagnostic status. The apparent c-statistic will be calculated and adjusted for optimism using bootstrapping^16^ with accompanying 95% CIs. Sensitivity will be calculated in each group at cut-offs of 95%, 99% and 99.9% specificity with accompanying 95% CIs. Accuracy of the ToO and cancer stage predictions will be calculated in the model training and validation groups with accompanying 95% CIs.

These analyses will be repeated, stratified by early stage (stage I or II) and late stage (stage III or IV) cancer; ToO; symptomatic controls and healthy volunteers; age band at recruitment; biological sex at birth; availability of screening programme for cancer (screening test available (breast, cervical, colorectal and lung) or not available). All analyses will be conducted in R V.4.4.2 (or higher) or STATA V.17 (or higher).

### Ethics and dissemination

This study is sponsored by University Hospitals Southampton NHS Foundation Trust and has been approved by the Health Research Authority and Health and Care Research West Midlands (24/WM/0234). This study is funded jointly by the National Institute for Health Research and the Office for Life Sciences (ref NIHR207538). SCTU is committed to the responsible sharing of clinical study data and samples with the wider research community. Data and sample access are administered through the SCTU Data and Sample Release Committee, which will consider requests once the final analysis has been published.

Results will be presented at scientific meetings and published in international peer-reviewed journals. Lay summaries of study progress and findings will be published on SCTU’s website.

### Patient and public involvement

This study benefits from two funded PPI coapplicants who have lived experience of cancer, have extensive experience in providing a patient’s perspective in cancer research and an interest in equality, diversity and inclusion. They have contributed to the conception and development of the study and will be advising on the content and tone of patient-facing materials. They will also lead the public dissemination strategy and participate in all regular study management group meetings.

## Discussion

This study will determine the diagnostic accuracy of machine learning techniques using the Enlighten test to detect cancer. This will be achieved by running the assay in a group of recently diagnosed cancer patients, patients who are symptomatic but cancer was ruled out, and participants with no symptoms or cancer diagnosis. Utility in guiding cancer diagnosis will also be assessed by determining the accuracy of the predicted ToO and stage of cancer. Cancer diagnosis will be collected and updated via local EHRs across five secondary care sites in the south of England. The potential applicability of the machine learning algorithms in future research will be assessed through a held-out dataset.

While this study focuses on 10 sites of cancer (bladder, breast, colorectal, lung, melanoma, oesophageal, ovarian, pancreatic, prostate and renal), additional ToO may be added, which will require model retraining in future research. This will reduce the risk of false results where a tested individual has a cancer that is not covered by the algorithm, which can delay the time to diagnosis. To improve the proportion of cancers detected early, a substantial number of cancer patients diagnosed at stage I or II must be recruited into the study. The research team have reviewed the throughput of recruitment sites in previous years, and recruitment will be monitored and potentially tailored to ensure that these requirements are met.

Theoretically, amino acids will improve the sensitivity for MCEDs to detect early-stage cancers in comparison to the more commonly used cfDNA and ctDNA techniques. If the Enlighten test does perform in this regard, then it could be used as a triage tool prior to expensive diagnostic imaging for individuals suspected of cancer. A reliable rule-in tool for cancer detection would reduce the overall demand for diagnostic imaging. This would, in turn, allow for individuals with a lower pretest probability of cancer to be tested, therefore increasing the rate of cancers detected early.

If the results from this study are promising, then the aim of future research will be to demonstrate an improvement in early-stage cancer detection balanced against the harms of overdiagnosis and cost in a randomised controlled trial. The feasibility of progressing to this stage depends on the recruitment success for all combinations of cancer ToO and stages, and the clinical acceptability of a 10 cancer MCED versus developing the MCED across further cancer ToO.

## Supplementary material

10.1136/bmjopen-2025-108220 online supplemental file 1
