# Supplementary material for: Multicancer early detection in a cohort of patients with confirmed and suspected cancer by measuring plasma amino acid cross sections with the Enlighten test: MODERNISED protocol
Source: BMJ Open. 2025 Nov 4;15(11):e108220. doi: 10.1136/bmjopen-2025-108220 (PMC12588033; doi:10.1136/bmjopen-2025-108220)
Supplement: Supplementary file 2 [file bmjopen-2025-108220.reviewer_comments.pdf]

PEER REVIEW HISTORY

BMJ Open publishes all reviews undertaken for accepted manuscripts. Reviewers are asked to complete a checklist review form and are provided with free text boxes to elaborate on their assessment. These free text comments are reproduced below.

ARTICLE DETAILS

Title (Provisional)

MODERNISED protocol: multi-cancer early detection in a cohort of patients with confirmed and suspected cancer by measuring plasma amino acid cross sections with the Enlighten test

Authors

Wilding, Sam; Goss, Victoria; Sukdao, Wesley; Hamady, Zaed; Lord, Joanne; Coleman, Adam; Pointer, Catherine; Walters, Jocelyn; Herbert, Will; Mclaughlin, Katy; Waugh, Robert; Irvine, Nicola; Oliver, Tom; Soulsby, Irene; Hooper, Julie; Crabb, Simon J.; Griffiths, Gareth; Yates, Emma; Davies, Andrew

---

VERSION 1 - REVIEW

Reviewer 1

Name Weitzer, Friedrich

Affiliation Medical University of Graz, Dep. of Radiology, Div. of Nuclear Medicine

Date 11-Aug-2025

COI None

Well depicted and sound study protocol, no major flaws are present. Updates are needed if interim analyses reveal interesting facts or indicate significant changes in protocol.

Reviewer 2

Name Fachiroh, Jajah

Affiliation Universitas Gadjah Mada, Histoogy and Cell Biology

Date 13-Sep-2025

COI None

MODERNISED protocol describe study to train and validate machine learning classifier of amino acid classifier using Enlighten test aiming to screen multiple cancers.

Study is well described with some parts need to be explained further in the text

1. Date of study

2. I found the registration of the study, please include in the method section

3. How the study describe symptoms related to 10 different cancers, that was overruled by the healthcare system, what are the symtoms? (table 1 point 5)

4. How does the study reach the potential participants?

5. How does the study recruit the potential participants (is it through clinical pathway)?

6. Please add data management plan, including data safety and security, as well as potential longterm use of specimen and data.

7. There are 3 potential randomisation of participants (page 9, line 12-16), please explain the reason/ justify.

8. Information sheet aims for cancer patients, any difference in explanation for undiagnosed potential cancer and healthy? please include in the revised information

sheet

VERSION 1 - AUTHOR RESPONSE

Reviewer: 1
Dr. Friedrich Weitzer, Medical University of Graz
Comments to the Author:
Well depicted and sound study protocol, no major flaws are present. Updates are needed if interim analyses reveal interesting facts or indicate significant changes in protocol.

Thank you for your kind comments.

Reviewer: 2
Dr. Jajah Fachiroh, Universitas Gadjah Mada
Comments to the Author:
MODERNISED protocol describe study to train and validate machine learning classifier of amino acid classifier using Enlighten test aiming to screen multiple cancers.

Study is well described with some parts need to be explained further in the text

Thank you for your summary and the comments below.

1. Date of study

The sentence "Recruitment began in January 2025." has been added to clarify this.

2. I found the registration of the study, please include in the method section

The ISRCTN number has been included in the method section.

3. How the study describe symptoms related to 10 different cancers, that was overruled by the healthcare system, what are the symtoms? (table 1 point 5)

The symptoms for each cancer is now attached as an appendix.

4. How does the study reach the potential participants?
5. How does the study recruit the potential participants (is it through clinical pathway)?

New wording has been added under the 'study participants' sub-heading to provide further information:

The first cohort, 'cancer cases', will consist of 1,000 adults recently diagnosed with one of the ten cancers, recruited from secondary care sites prior to treatment. The second cohort, 'symptomatic controls', will consist of 250 adults either currently or previously under investigation for one of the ten cancers, recruited from secondary care sites, who are found to be cancer free following normal diagnostic pathways. Participants in this cohort who are found to have cancer during follow-up will be moved to the cancer cases cohort. **These participants will be reached via screening of clinic lists, and approached during routine hospital visits for symptom investigation, or by phonecall to invite them to attend an enrolment visit**. The third cohort, 'healthy volunteers' will consist of 100 adults with no known cancer and no active symptoms indicative of cancer, recruited from volunteer groups at secondary care sites, or University and hospital staff if recruitment is slower than anticipated. **These participants will be reached by advertisement of the study in email newsletters, and invited to attend recruitment clinics.** Participants in this cohort who are found to have cancer during follow-up will be moved to the cancer cases cohort. Full inclusion and exclusion criteria can be found in Table 1. The study workflow is outlined in Figure 1.

6. Please add data management plan, including data safety and security, as well as potential longterm use of specimen and data.

We have included the following sentence on data safety and security:

"Pseudonymised data will then be entered into Medidata's Rave EDC, which can then only be accessed by allocated staff at Southampton Clinical Trials Unit."

The authors feel that long-term use of specimens and data is already covered in the manuscript under the 'Ethics and dissemination' section:

"SCTU is committed to the responsible sharing of clinical study data and samples with the wider research community. Data and sample access is administered through the SCTU Data and Sample Release Committee, who will consider requests once the final analysis has been published."

7. There are 3 potential randomisation of participants (page 9, line 12-16), please explain the reason/ justify.

This sentence has been expanded to justify (in bold):

Randomisation will therefore only occur at three occasions during the study **to reduce the impact on imbalance between building and validation sets**.

8. Information sheet aims for cancer patients, any difference in explanation for undiagnosed potential cancer and healthy? please include in the revised information sheet

Three patient information sheets (PIS) are attached to this submission, one for confirmed cancer (A), one for suspected cancer (B) and one for healthy volunteers (C).
